# Association of the IP3R to STIM1 provides a reduced intraluminal calcium microenvironment, resulting in enhanced store-operated calcium entry

**DOI:** 10.1038/s41598-018-31621-0

**Published:** 2018-09-05

**Authors:** Alicia Sampieri, Karla Santoyo, Alexander Asanov, Luis Vaca

**Affiliations:** 1 Departamento de Biologia Celular y del Desarrollo, Instituto de Fisiología Celular. Universidad Nacional Autonoma de México, Ciudad de México, Mexico; 2 TIRFLabs Inc, 106 Grendon Place, Cary, NC USA

## Abstract

The involvement of inositol trisphosphate receptor (IP3R) in modulating store-operated calcium entry (SOCE) was established many years ago. Nevertheless, the molecular mechanism responsible for this observation has not been elucidated to this date. In the present study we show that IP3R associates to STIM1 upon depletion of the endoplasmic reticulum (ER) by activation of the inositol trisphosphate signaling cascade via G-protein coupled receptors. IP3R-STIM1 association results in enhanced STIM1 puncta formation and larger Orai-mediated whole-cell currents as well as increased calcium influx. Depleting the ER with a calcium ATPase inhibitor (thapsigargin, TG) does not induce IP3R-STIM1 association, indicating that this association requires an active IP3R. The IP3R-STIM1 association is only observed after IP3R activation, as evidenced by FRET experiments and co-immunoprecipitation assays. ER intraluminal calcium measurements using Mag-Fluo-4 showed enhanced calcium depletion when IP3R is overexpressed. A STIM1-GCaMP fusion protein indicates that STIM1 detects lower calcium concentrations near its EF-hand domain when IP3R is overexpressed when compared with the fluorescence reported by a GCaMP homogenously distributed in the ER lumen (ER-GCaMP). All these data together strongly suggest that activation of inositol trisphosphate signaling cascade induces the formation of the IP3R-STIM1 complex. The activated IP3R provides a reduced intraluminal calcium microenvironment near STIM1, resulting in enhanced activation of Orai currents and SOCE.

## Introduction

Store-operated calcium entry (SOCE) is a conserved mechanism by which the depletion of the endoplasmic reticulum (ER) is conveyed to calcium-permeable channels at the plasma membrane (PM), triggering calcium influx from the extracellular space and into the cell cytosol^1^. A physiological mechanism responsible for the activation of SOCE results from the stimulation of G-protein coupled receptors associated to the inositol-triphosphate (IP3) and phospholipase C cascade, resulting in the release of calcium from ER, via the IP3 receptor (IP3R)^2^. The emptying of the ER initiates the dissociation of ER luminal calcium from the EF-hand of the stromal interacting molecule (STIM1). This protein is a sensor of the calcium content in the ER^3^.

The dissociation of calcium from the EF-hand in STIM1, results in the oligomerization of this protein and the formation of the so-called STIM1 puncta^4^. Others and we have identified several proteins recruited to STIM1 puncta upon ER depletion. Thus the puncta appears to be a macromolecular multi protein-signaling complex responsible for facilitating SOCE and calcium homeostasis. Some of the proteins recruited to the puncta include the calcium ATPase (SERCA)^5^, SARAF^6^, junctate^7^ and several other^8^.

The IP3R plays a crucial role in the exit of calcium from the ER upon activation of inositol-triphosphate (IP3) and phospholipase C cascade^2,9^. Initial studies have shown that IP3R also modulates SOCE and calcium influx^10^. A recent study shows that STIM1 can also modulate IP3R activity^11^. Furthermore, IP3R clusters optimally placed near STIM1 modulate calcium entry^12^.

The molecular mechanism by which IP3R modulates SOCE has not been completely elucidated. Initial studies indicated that a direct coupling between IP3R and calcium influx channels was responsible for the activation of SOCE^10^. After the discovery of STIM1, less attention was placed on exploring further these initial findings. Nevertheless, a wealth of published reports show a strong link between SOCE and IP3R activity^13–15^.

In the present study we show that IP3R is recruited to STIM1 puncta upon depletion of the ER via activation of G-protein coupled receptors associated to the inositol-triphosphate (IP3) and phospholipase C signaling cascade. The association of STIM1 and IP3R at the puncta is supported by co-localization confocal microscopy studies, Förster resonance energy transfer (FRET), total internal reflection microscopy (TIRFM) and co-immuniprecipitation assays.

Overexpression of IP3R results in enhanced ER depletion (as assessed by Mag-Fluo-4 measurements), larger diameter puncta formation and increased Orai whole-cell currents as well as enhanced calcium influx. A STIM1-GCaMP fusion protein designed to assess calcium content near the EF-hand in STIM1 reports faster and stronger reduction of calcium content near STIM1 after activation of IP3R when compared with a GCaMP using an ER retention signal (ER-GCaMP), which is homogenously distributed in the ER lumen.

All these results suggest a model in which activation of IP3R via agonists results in the recruitment of the IP3R to the STIM1 puncta and its association to STIM1. The formation of the STIM1-IP3R complex facilitates a reduced calcium microenvironment near the EF-hand from STIM1, which enhances puncta formation and activation of Orai channels at the plasma membrane.

## Results

### IP3R is recruited to STIM1 puncta upon depletion of the ER via activation of G-protein coupled receptors

**Figure 1:**
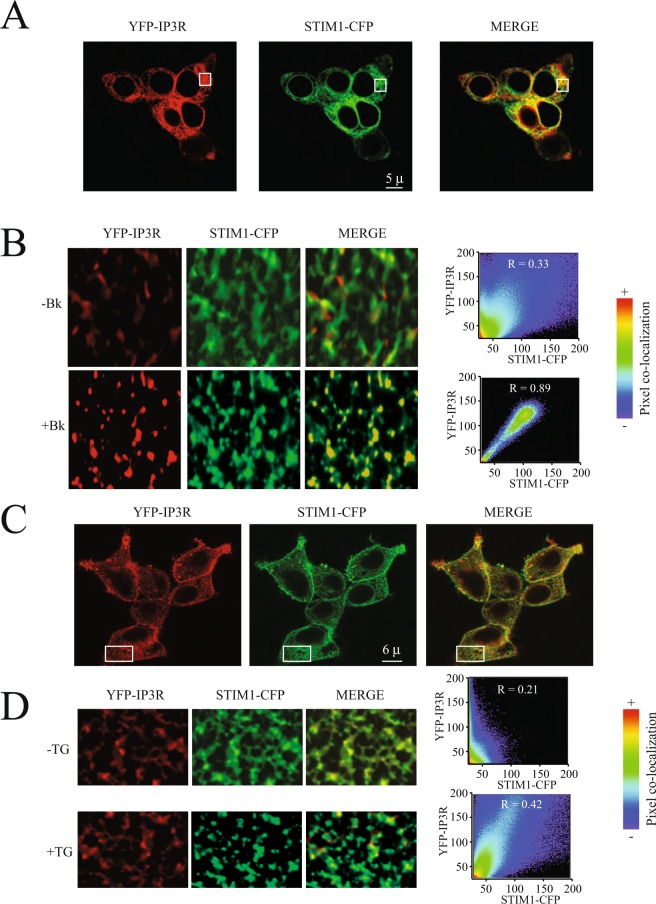
Active IP3R are recruited to the STIM1 puncta upon activation. (**A**) Typical confocal microscopy images illustrating the localization of the YFP-IP3R (shown in red) and STIM1-CFP (shown in green) at the endoplasmic reticulum in cells stimulated with bradykinin (Bk). (**B**) Amplification of the areas shown in A with a white rectangle to show in greater details the puncta formation for YFP-IP3R (shown in red) and STIM1-CFP (shown in green). To the right are the co-localization panels obtained with Imaris before and after bradykinin (Bk) stimulation. (**C**) Typical confocal microscopy images illustrating the localization of the YFP-IP3R (shown in red) and STIM1-CFP (shown in green) at the endoplasmic reticulum in cells stimulated with thapsigargin (TG). (**D**) Amplification of the areas shown in A with a white rectangle to show in greater details the puncta formation for YFP-IP3R (shown in red) and STIM1-CFP (shown in green). To the right are the co-localization panels obtained with Imaris before and after thapsigargin (TG) stimulation. R represents the Pearson correlation coefficient. Co-localization pixel by pixel was mapped to the pseudo color scale illustrated to the right from 0 (blue) to 1 (red). Scales show 5 microns (μ) for panel A and 6 microns (μ) for panel B.

Expression of recombinant YFP-IP3R (IP3R type 1 or IP3R1) in combination with STIM1-CFP in HEK293 cells shows that both proteins reside in the endoplasmic reticulum, as expected (Fig. 1A). Activation of IP3R via stimulation of G-protein coupled receptors associated to the inositol-triphosphate (IP3) and phospholipase C cascade results in STIM1 puncta formation (Fig. 1B). Most surprisingly, IP3R are recruited to the puncta and strong co-localization is observed between YFP-IP3R and STIM1-CFP after bradykinin stimulation (Fig. 1B, Supplementary Video 1). This strong co-localization (Pearson coefficient = 0.89 ± 0.12, n = 45) is evident with several agonists that activate G-protein coupled receptors associated to the inositol-triphosphate (IP3) and phospholipase C cascade. Using the calcium ATPase selective inhibitor thapsigargin (TG) results in reduced co-localization (Pearson coefficient = 0.42 ± 0.09, n = 38). Furthermore, IP3R does not appear to form puncta after TG stimulation, whereas STIM1-CFP forms a typical puncta (Fig. 1D). These results strongly suggest that IP3R activation is required for this receptor to form part of the puncta complex.

### The formation of the IP3R-STIM1 complex enhances whole-cell currents and calcium influx

**Figure 2:**
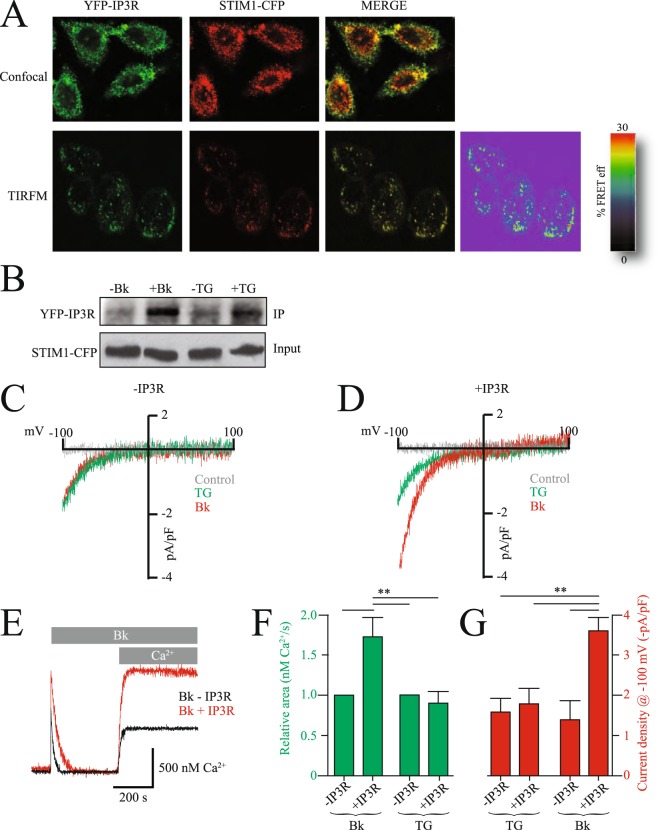
IP3R overexpression enhances SOCE and Orai1 currents. (**A**) Confocal (upper panel) and TIRFM (lower panel) co-localization studies for YFP-IP3R and STIM1-CFP. In TIRFM mode Förster Resonance Energy Transfer (FRET) experiments were conducted to assess the interactions between YFP-IP3R and STIM1-CFP. The right panel shows the percentage FRET efficiency (FRET eff) between the two fluorescent proteins. (**B**) Co-immunoprecipitation analysis for YFP-IP3R and STIM1-CFP before and after stimulation with thapsigargin (TG) or bradykinin (Bk). STIM1-CFP was used as bait (input). To reduce the size of the image and make it fit in the figure, bands were cutout from original gels, to see original gels please refer to Supp. Fig. 3. (**C**) Whole-cell patch clamp experiments with control cells (left panel) and cells overexpressing IP3R (right panel). Control (gray line) shows the currents before agonist application, in red is Bk and in green TG. Whole-cell were elicited with a ramp from 100 mV to +100 mV. (**E**) Fura-2 ratiometric calcium measurements in cell populations in control cells (black) and cells overexpressing the IP3R (red). (**F**) Measurements of relative area under the curve for the calcium measurements (green) and current density for whole-cell experiments (red) under the different conditions illustrated in the panel. Values are mean ± standard deviations from at least 30 independent measurements.

Because co-localization studies are restricted to the light diffraction limit, we conducted Förster resonance energy transfer (FRET), which provides a spatial resolution better than 10 nm. Basal FRET signal between YFP-IP3R and STIM1-CFP was 9 ± 5% FRET efficiency, which was indistinguishable from the FRET signal obtained with soluble forms of YFP and CFP (8.5 ± 4% FRET efficiency). FRET signal was significantly increased upon bradykinin stimulation to 24 ± 4% FRET efficiency (Fig. 2A). This FRET signal was obtained in total internal reflection fluorescence microscopy (TIRFM) mode, indicating that the FRET signal occurred in regions near the plasma membrane (less than 100 nm form the glass coverslip where cells are platted).

To confirm the bradykinin induced association of YFP-IP3R and STIM1-CFP using alternative methodologies, we conducted co-immmunoprecipitation (co-IPs) studies with both proteins. co-IPs provided further evidence of the association of YFP-IP3R and STIM1-CFP after activation of G-protein coupled receptors associated to the inositol-triphosphate (IP3) and phospholipase C cascade (Fig. 2B). Under resting conditions no co-IP was observed between YFP-IP3R and STIM1-CFP, but after bradykinin stimulation both proteins were co-IP (Fig. 2B). co-IP was not observed with thapsigargin stimulation (Fig. 2B and Supp. Fig. 1 for complete blots).

To evaluate the effect of IP3R-STIM1 complex formation on SOCE, we conducted whole-cell patch clamp electrophysiology experiments measuring endogenous Orai currents (Fig. 2C,D). First whole-cell currents time courses were evaluated to identify the times at which the currents reached maximum (steady state) values for agonists as well as thapsigargin stimulations (Supp. Fig. 2). Under control conditions whole-cell current density activated by bradykinin and TG was not significantly different (Fig. 2). However, whole-cell current density was greatly enhanced in cells overexpressing the IP3R only when stimulated with bradykinin but not with TG (Fig. 2D). Intracellular calcium measurements with control cells and cells overexpressing the IP3R further confirm the increment in calcium influx when stimulated with bradykinin but not with TG (Fig. 2E–G). Both whole-cell current density and intracellular calcium were increased about two fold in cells overexpressing IP3R (Fig. 2F,G). This observation was not exclusive for bradykinin, as similar results were obtained using histamine and carbachol, selective agonists of the histamine and acetylcholine receptors, respectively (Supp. Fig. 3). The endogenous whole-cell currents and intracellular calcium measurements stimulated with agonists were supported mainly by Orai1 channels, as concluded from RNA interference (RNAi) experiments directed to knock down Orai1 (Supp. Fig. 4). RNAi reduced the endogenous whole-cell currents by approximately 75% and calcium measurements by 60% (Supp. Fig. 4). We cannot discard that a different ionic channel may produce the remaining currents or may be the result of partial Orai1 knock down (no RNAi experiment is 100% effective).

### Overexpression of the IP3R increases depletion of the ER

**Figure 3:**
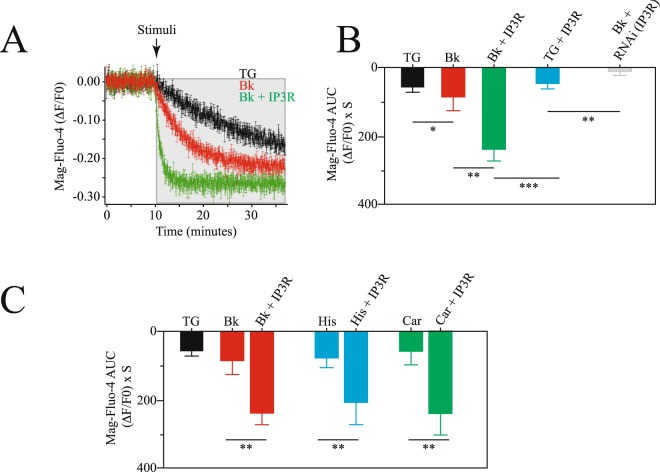
Overexpression of the IP3R induces a faster depletion of the endoplasmic reticulum. (**A**) Mean ± standard deviation of at least 25 independent measurements in cell populations of intraluminal ER calcium using Mag-Fluo-4 (Material and Methods). Data shows the fluorescence changes over time of corrected data (background subtraction, Material and Methods). (**B**) Area under the curve (AUC) of the Mag-Fluo-4 fluorescence changes under the following conditions: after TG stimulation (black), with bradykinin stimulation (red), bradykinin stimulation in cells overexpressing IP3R (green), TG in cells overexpressing IP3R (light blue) and bradykinin stimulation in cells with RNAi to reduce IP3R (gray). (C) Area under the curve (AUC) for Mag-Fluo-4 fluorescence using different agonists coupled to the IP3 cascade. Bradykinin stimulation in control cells and cells overexpressing IP3R (red), histamine stimulation in control cells and cells overexpressing IP3R (light blue) and carbachol stimulation in control cells and cells overexpressing IP3R (green). Data shows mean ± standard deviation of at least 25 independent measurements. Significance set at ***p < 0.001, **p < 0.01 or *p < 0.05.

Since the IP3R is indeed an ER calcium release channel, we decided to explore the effect of the overexpression of this receptor on the dynamics of ER calcium content using the calcium indicator Mag-Fluo-4 (Fig. 3). Mag-Fluo-4 is an analog of fluo-4 with a Kd for Mg^2+^ of 4.7 mM and a Kd for Ca^2+^ of 22 µM. Because it accumulates in the ER, it has been extensible used to monitor ER calcium content changes in real time^16^.

Cells overexpressing the IP3R showed faster reductions in intraluminal ER calcium content with agonists coupled to the inositol-triphosphate (IP3) and phospholipase C cascade (Fig. 3A,B). Comparing three different agonists with thapsigargin it was evident that the greater effect was observed in cells overexpressing the IP3R (Fig. 3C). These results confirm previous observations showing the role of the IP3R as an ER calcium release channel^9^.

### Overexpression increases puncta diameter

**Figure 4:**
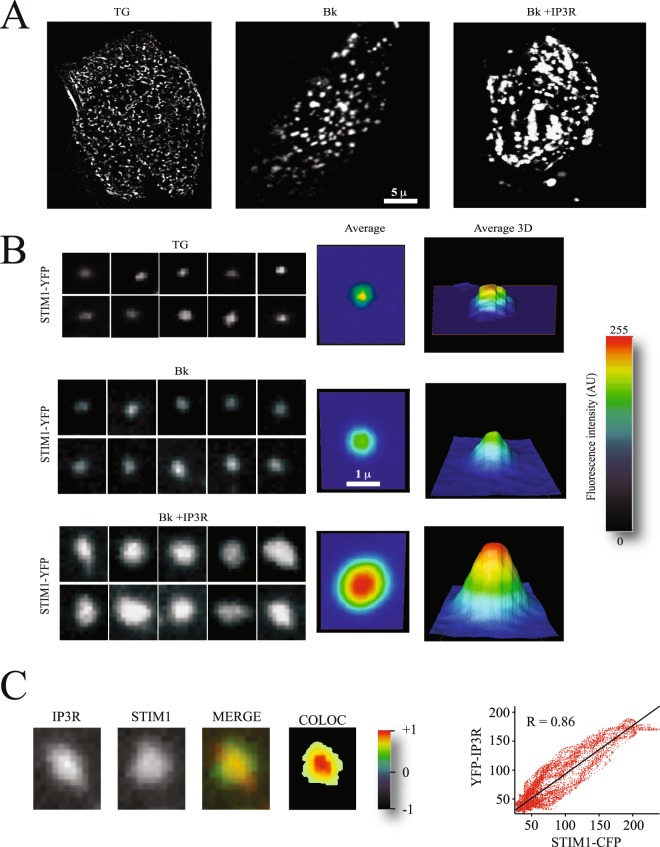
Overexpression of the IP3R enhances STIM1 puncta formation. (**A**) Total internal reflection microscopy (TIRFM) images of STIM1-CFP forming puncta. Each panel shows a single cell. Scale shows 5 microns (μ). (**B**) Examples of individual puncta obtained from cells like those shown in (**A**). Each panel shows a single punctum. Middle panel shows the average of at least 120 individual puncta obtained from at least 15 independent cells. The panel to the right shows the 3D projection of the average puncta for each condition. The height shows the intensity in pseudo color scale (shown to the extreme right). The conditons explored are puncta formation induced by thapsigargin (TG), bradykinin (Bk) and Bk in cells overexpressing the IP3R. (**C**) Example of a single puncta, illustrating the co-localization of STIM1-CFP and YFP-IP3R. The right panel shows a pseudo color scale of the pixel co-localization according to Pearson coefficients (material and Methods).

Because IP3R activation recruits this receptor to the puncta, we explored in greater detail the effects of the inclusion of this protein in the puncta. Most interestingly, IP3R overexpression resulted in larger diameter puncta in cells stimulated with agonists coupled to the inositol-triphosphate (IP3) and phospholipase C cascade but not in cells stimulated with TG (Fig. 4A,B). The average puncta diameter obtained after measuring at least 100 independent puncta was about 3–4 times greater than that obtained in control cells or cells overexpressing the IP3R but stimulated with TG (Fig. 4B). As previously shown, YFP-IP3R and STIM1-CFP co-localize in the puncta with a Pearson coefficient of 0.86 ± 0.09 (Fig. 4C). The centers of the puncta showed the greater co-localization coefficients (Fig. 4C). These results show that overexpression and activation of IP3R results in its recruitment to the STIM1 puncta and significantly increases the puncta diameter.

### IP3R provides a calcium-reduced microenvironment to enhance STIM1 puncta formation

To further explore the molecular mechanism by which recruitment of the IP3R to the puncta results in puncta with greater diameters, we designed a strategy to monitor calcium changes near the EF hand from STIM1. Since the main constituent of the puncta is STIM1, a safe assumption is that puncta with larger diameters reflect more STIM1 oligomerization. Since STIM1 oligomerization is driven by the depletion of the ER, which results in the dissociation of calcium from the EF hand from STIM1; one possible mechanism for IP3R to enhance STIM1-oligomerization could be by increasing the depletion of the ER. Because IP3R closely associates to STIM1 after its activation (as evidenced by the FRET and co-IP experiments presented above), we speculated that the mechanism by which IP3R enhances puncta formation is by providing a reduced calcium microenvironment near the EF hand from STIM1.

**Figure 5:**
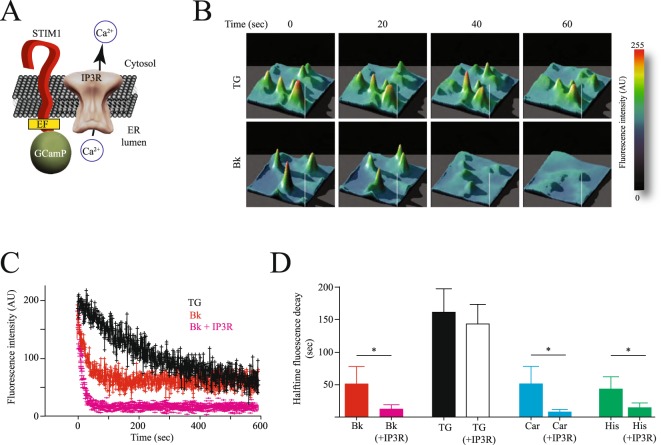
IP3R provides a low calcium microenvironment near the EF hand from STIM1 to induce enhanced STIM1 oligomerization. (**A**) Cartoon illustrating the reporter fusion protein between STIM1 and the calcium GECI GCaMP 3 (STIM1-GCaMP). Notice that the GCaMP is fused at the amino terminus of STIM1, which is located inside the endoplasmic reticulum (ER). GCaMP is position a few amino acids after the EF hand from STIM1. The idea was to use GCaMP to monitor local changes in calcium near the STIM1 EF hand. The IP3R is positioned near STIM1, in agreement with data from FRET, TIRFM and co-immunoprecipitation. (**B**) Representative 3D plots of the changes in fluorescence reported by STIM1-GCaMP fusion protein over time. Examples show the time course of fluorescence reduction in cells stimulated with thapsigargin (TG) or bradykinin (Bk). (**C**) Mean ± standard deviation of changes in fluorescence reported by STIM1-GCaMP fusion protein over time. Data represents measurements from individual puncta obtained from at least 46 independent cells for each condition. In black is shown cells stimulated with TG, in red cells stimulated with Bk and in pink cells overexpressing the IP3R and stimulated with Bk. (**D**) Half time florescence decay reported by the STIM1-GCaMP fusion protein obtained from experiments like those shown in (**C**). conditions are: Bk in control cells (red) and cells overexpressing IP3R (pink), TG in control cells (black) and cells overexpressing IP3R (white), carbachol (Car) in control cells and cells overexpressing IP3R (light blue) and histamine (His) in control cells and cells overexpressing IP3R (green). Significance set at **p < 0.01 or *p < 0.05.

To explore this hypothesis we produced a fusion protein of STIM1 and the genetically encoded low affinity variant of GCaMP 3 calcium indicator^17^. We fused GCaMP at the amino terminus of STIM1, in closed proximity to the amino acid region encoding the EF hand (Fig. 5A).

The GCaMP-STIM1 fusion reporter protein showed faster reduction in fluorescence (calcium concentration) in cells exposed to agonists coupled to the inositol-triphosphate (IP3) and phospholipase C cascade, but not to TG (Fig. 5B, Supplementary Video 2). The time course of reduction in calcium near the EF hand showed a 3 fold faster reduction in cells overexpressing the IP3R when compared to control cells (Fig. 5C,D). This effect was evident when cells were stimulated with bradykinin, histamine and carbachol (Fig. 5D). Cells overexpressing the IP3R but stimulated with TG did not show the accelerated reduction in calcium observed in cells stimulated with agonists (Fig. 5D).

**Figure 6:**
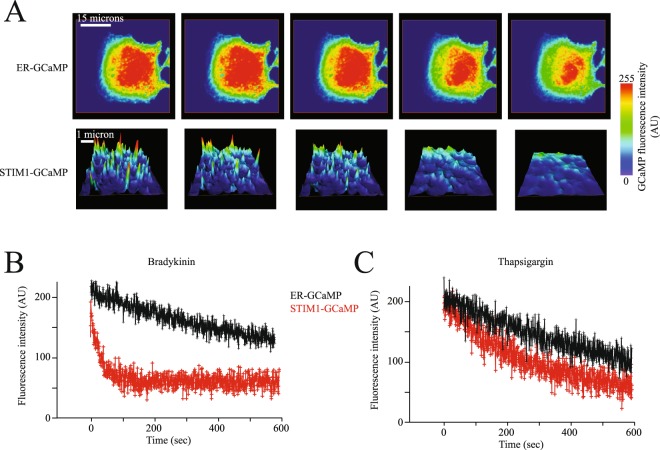
STIM1 senses a reduced calcium microenvironment when IP3R is nearby. (**A**) representative confocal microscopy images of the calcium dynamics at the ER lumen detected by the ER-GCaMP (upper panels) and STIM1-GCaMP (lower panels) calcium sensors. Focal plane was placed at the bottom of the cell, close to the interface with the coverslip to visualize the ER in proximity to the plasma membrane. Notice that fluorescence reported by the ER-GCaMP sensor is homogenously distributed throughout the ER lumen. Upper panels in **A** show the entire ER from a single cell while lower panels show images of a region of interest inside a cell. For clarity lower panels are shown as 3D height plots to more clearly illustrate the individual puncta. (**B**) Time course of florescence reported by the ER-GCaMP sensor (black) and the STIM1-GCaMP sensor (red) in response to bradykinin (**B**) and thapsigargin (**C**). Data shows the mean ± standard deviation from at least 32 cells obtained from 5 independent transfections. For ER-GCaMP the fluorescence was obtained from the entire ER while with the STIM1-GCaMP fluorescence was measured in individual puncta. Data with the STIM1-GCaMP sensor contains fluorescence from approximately 20–30 individual puncta from each cell. Statistical analysis of all points indicate that ER-GCaMP and STIM1-GCaMP are different at the value of p < 0.001 with bradykinin stimulation, while no significant difference was obtained with thapsigargin stimulation.

To compare local calcium microdomains near STIM1 (via the STIM1-GCaMP sensor) with general ER luminal calcium changes, we produced a low affinity variant of GCaMP 3 calcium indicator with an ER retention signal (ER-GCaMP, see Material and Methods for details). Using the ER-GCaMP we observed a homogenous distribution of fluorescence at the ER (Fig. 6A, upper panels). Both agonists and TG stimulation resulted in a reduction in fluorescence as reported by the ER-GCaMP sensor (Fig. 6A) compared to fluorescence in puncta reported by the STIM1-GCaMP sensor (Fig. 6B). Bradykinin stimulation produced a fast reduction in fluorescence reported by the STIM1-GCaMP sensor, whereas the ER-GCaMP sensor reported a slower fluorescence reduction under the same conditions (Fig. 6B). TG stimulation produced indistinguishable time courses in fluorescence reduction reported by STIM1-GCaMP and ER-GCaMP (Fig. 6C, see also Supplementary Video 3). All these results strongly suggest that STIM1 is sensing a reduced calcium microenvironment near its EF-hand when the ER is depleted by agonists but not by TG. Because the ER-GCaMP sensor is not associated to STIM1, this sensor does not detect the localized calcium changes in the microenvironment near STIM1 EF hand; only the STIM1-GCaMP sensor reports such changes.

**Figure 7:**
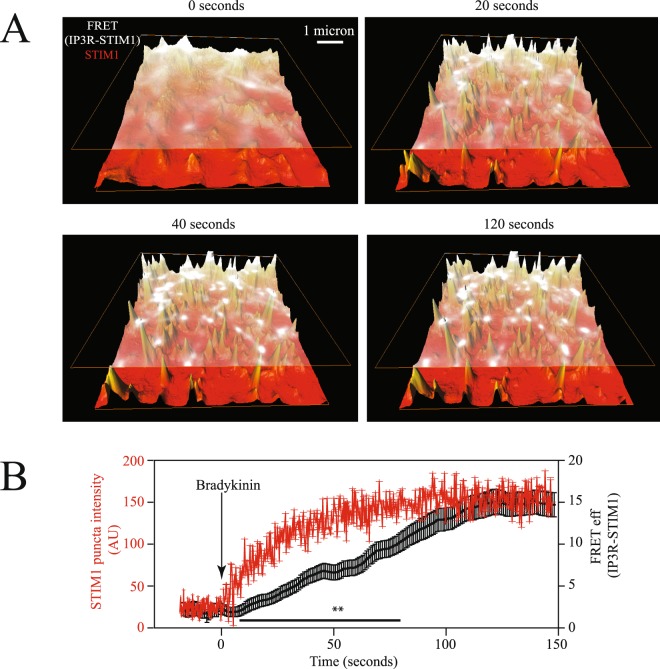
IP3R is recruited into STIM1 puncta at a later time. Time courses of STIM1-CFP fluorescence (shown in red in 3D height plot) and FRET signal between YFP-IP3R/STIM1-CFP (illustrated in white) upon bradykinin stimulation. (**A**) Shows fluorescence of STIM1-CFP and FRET signal between IP3R and STIM1 in perspective view. For illustration purposes the FRET signal is placed above the STIM1 fluorescence. (**B**) Time courses of fluorescence intensity and FRET between STIM1-CFP and YFP-IP3R under control conditions and after bradykinin stimulation (shown with an arrow). Data shows the mean ± standard deviation from at least 43 cells obtained from 6 independent transfections. Data with the STIM1-CFP contains fluorescence from approximately 20–30 individual puncta from each cell. FRET data represents the FRET signal obtained at each puncta (see Figs 2 and 4 for details on how FRET at each puncta was assessed). Statistical analysis of all points indicate that FRET data and STIM1-CFP fluorescence are different between the time points of 10–75 seconds at the value of p < 0.01 Illustrated with **. For an example of typical time courses please refer to Supplementary Video 4).

**Figure 8:**
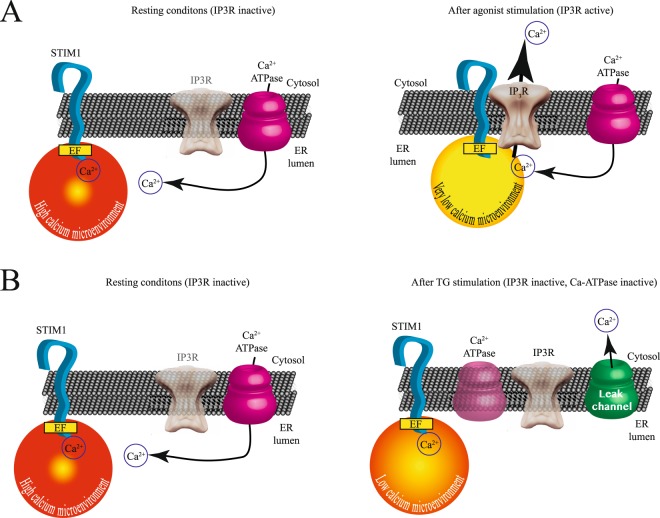
A model depicting the role of active IP3R in generating a low calcium microenvironment near STIM1 EF hand to enhance puncta formation and SOCE. (**A**) The left panel shows a cartoon illustrating the resting conditions in the cell with high intraluminal ER calcium (stores full). Under these conditions the IP3R is inactive (semitransparent). The right panel shows the conditions after agonist stimulation with an active IP3R. Under these conditions the IP3R is recruited to the puncta near STIM1, facilitating the clearing of intraluminal calcium and generating a reduced calcium microenvironment near STIM1 EF hand. (**B**) The left panel shows a cartoon illustrating the resting conditions in the cell with high intraluminal ER calcium (stores full). The right panel shows the conditions where TG is used to block the calcium ATPase. This results in a reduction of intraluminal calcium. However, under these conditions IP3R does not migrate to the puncta and with an inactive IP3R a higher calcium microenvironment near STIM1 EF hand is produced. This results in less SOCE and thus smaller Orai currents.

To further evaluate the incorporation of IP3R to the STIM1 puncta, we performed fluorescence time courses in cells expressing YFP-IP3R and STIM1-CFP (Fig. 7). As illustrated in the Fig. 7, the FRET between STIM1-CFP and YFP-IP3R occurred after the STIM1 puncta was clearly visible; indicating that the IP3R is recruited to the STIM1 puncta after puncta formation is initiated (Fig. 7C). These results indicate that puncta formation is the initial step and the IP3R incorporates later (see also Supplementary Video 4). Figure 8 depicts a cartoon illustrating the possible mechanism responsible for generating an intraluminal calcium microenvironment, which stimulates STIM1 puncta formation and enhanced SOCE and Orai1 currents. Under resting conditions IP3R is inactive and away from STIM1 (Fig. 8A). Agonist stimulation results in puncta formation and the recruitment of IP3R to the puncta (Fig. 8A). Under these conditions very low calcium microenvironment near STIM1 is generated by the IP3R. TG stimulation does not activate IP3R and thus the receptor is not recruited to the STIM1 puncta (Fig. 8B). Under these conditions only, a reduced calcium microenvironment is generated near SITM1.

## Discussion

The role of IP3R as the main calcium release channel in the endoplasmic reticulum from the majority of cells is well established^9,18^. However IP3R plays different roles in calcium homeostasis, most importantly its role as modulator of calcium influx from the extracellular space^14,19^. This later role is less well studied and understood. Initial studies indicated a direct activation of calcium channels at the plasma membrane by the IP3R^10^. The more recent identification of STIM1 as the ER calcium sensor and its role in the activation of Orai channels concentrated most of the studies on understanding the interactions between STIM1 and Orai. Nevertheless, it is clear that IP3R plays complex roles in modulating calcium influx, as illustrated by a wealth of studies published during the last 20 years.

Recent studies shows the modulation of IP3R by STIM1^11,12^. This modulation affects the amount of calcium release via IP3R, which in turn modulates SOCE by altering the degree of depletion of the ER. Super-resolution microscopy studies show that the calcium signal initiates with immobile IP3R located at the ER-PM junctions^12^. The ER-PM junction is formed upon activation of SOCE by the depletion of the ER, which triggers the oligomerization of STIM1 and its association to Orai channels at the PM. Although the aforementioned super-resolution microscopy study does not provide a molecular explanation for the IP3R immobile fraction, the results presented here strongly suggest that the IP3R is immobilized by its incorporation into the STIM1 puncta as part of a protein complex, which includes other members that are recruited to the puncta, such as the calcium ATPase^5^ and many other members of what we had named the store-operated calcium influx complex (SOCIC)^8,20^. Even though in the mentioned study they did not find significant co-localization between IP3R immobile fraction and STIM1, it is worth mentioning that a strong co-localization between this IP3R fraction and microtubules was observed^12^. It is feasible that the interactions observed in the present study between IP3R and STIM12 may be mediated by microtubules, after all STIM1 is a MT-plus-end-tracking protein involved in remodeling of the ER^21^, and STIM1 associates to the end binding protein 1 (EB1) to track microtubules^5,22^.

Furthermore, here we have shown that IP3R incorporates into nascent puncta following initial puncta formation. Thus the time at which IP3R-STIM1 association is evaluated is vital to find strong co-localization between both proteins (Fig. 7C). We conclude that these IP3R that get incorporated into the puncta is the immobile fraction described elsewhere^12^. Even more, our results strongly suggest that these immobilized IP3R fraction provide a reduced calcium microenvironment near STIM1 EF-hand, which facilitates puncta formation and results in greater Orai1 currents.

A previous study has shown that STIM1 associates to the IP3R1 and that this association mediates the agonist-stimulated calcium entry via Orai1 channels^23^. Our results are in agreement with this initial observation and provide further insights into the molecular mechanism responsible for this observation.

In the present study we show evidence indicating that the IP3R is recruited to the STIM1 puncta upon depletion of the ER by agonists coupled to the inositol-triphosphate (IP3) and phospholipase C cascade. Depletion of the ER using the calcium ATPase inhibitor thapsigargin (TG) does not induce recruitment of the IP3R, even though results in the depletion of the ER. Förster resonance energy transfer (FRET), total internal reflection microscopy (TIRFM) and co-immuniprecipitation assays all provide evidence of a direct association of active IP3R to STIM1 at the puncta. The translocation of the IP3R to the puncta and its proximity to STIM1 provides a low calcium microenvironment for STIM1 (detected by the EF hand domain from STIM1) that enhances puncta formation and conveys a greater signal for the activation of Oria1 channels at the plasma membrane. This enhanced signal results in larger whole-cell currents and increased calcium influx. We speculate that by controlling the amount of IP3R expressed, cells can fine-tune not only the depletion of the ER, but also the amount of SOCE activated after the depletion. The experimental evidence gathered here in combination with previous studies illustrating the modulation of IP3R by STIM1 strongly suggests a reciprocal modulation of both proteins by its interactions. One can only speculate how other proteins of the SOCIC may alter this reciprocal modulation of STIM1-IP3R, which is a topic that deserves future studies.

## Material and Methods

### Reagents

All salts were analytical grade from Sigma (Saint Luis, MO). Anti-STIM1 antibody S6072 and anti-GFP were purchased from Sigma (San Luis, MO). For IP3R we used the antibody from Abcam (ab5804), Abcam (Cambridge, MA). DAPI, Opti-MEM, Dulbecco’s medium (DMEM) and all restriction enzymes were purchased from Invitrogen (Carlsbad, CA. USA). STIM1 plasmid was a generous donation from Dr. Tobias Meyer (Stanford University). The type 1 IP3R was a generous donation from Dr. Katsuhiko Mikoshiba (RIKEN Brain Science Institute).

### Constructs

For simultaneous co-localization and FRET studies, the following fusion proteins were produced: YFP-IP3R and STIM1-CFP. For the construction of STIM1-GCaMP, we used a low affinity variant of GCaMP 3^17^ and remove its stop codon by PCR and then cloned in pBluescript II (Addgene, Cambridge, MA). The GCaMP was then cloned in frame with STIM1 at its 5′. The entire product was sequenced prior to using it in calcium measurements. For ER-GCaMP we used the same low affinity variant and fused to the calreticulin signal peptide (MLLSVPLLLGLLGLAVA) at the amino terminus of GCaMP3^17^. All nucleic acid constructs were fully sequenced after the last ligation procedure to ensure integrity and the correct reading frame.

### Cell culture and transfection

HEK293T cells were grown in DMEM supplemented with 10% (V/V) fetal bovine serum, 50 µg/ml penicillin, 50 µg/ml streptomycin and maintained at 37 °C in a humidified atmosphere of 5% CO_2_ and 95% air. For imaging experiments, one day prior to transfection with the plasmid of choice, the cells were plated onto 25 mm circular coverslips. Plasmids of YFP-IP3R and STIM1-CFP were transfected using lipofectamine 2000 (Thermo Fisher Scientific) following manufacturer instructions.

### Expression of the human bradykinin type 2 receptor

The clone form the human bradykinin type 2 receptor was transfected in HEK293 cells, since this cell line does not express this receptor^24,25^. For the other two agonists we used the endogenous histamine and acetylcholine receptors.

### RNA interference studies

HEK293T cells were transfected with a siRNA for Orai1 (SR313705) purchased from Origene (Rockville, MD). As negative control we used the universal scramble sequence provided by the manufacturer (RNAi-scramble). siRNAs were transfected as previously reported^26^. To improve RNA interference efficiency, cells were transfected twice. The second transfection was performed 3 days after the first one. Transfected cells were studied between 48–72 hours after the last transfection. The transfection efficiency of the siRNAs was determined using a fluorescently labeled siRNA as previously described^27^. In all cases over 60% of the cells were transfected, as evaluated after the second transfection.

### Real-time polymerase chain reaction (rPCR)

To assess the effectiveness of our RNA interference method, we quantified the mRNA levels using rPCR, for Oria1, as previously described^27^. Briefly, total RNA was purified from HEK293T cells transfected with a control siRNA or a siRNA for Orai1 (SR313705). For determination of IP3R mRNA levels we used oligonucleotides directed to amplify a region between 1021–1261 nucleotides (GenBank NM_001168272.1). For complementary DNA (cDNA) synthesis, 2.5 μg of total RNA was reverse transcribed using the SuperScript III RT (Invitrogen, Carlsbad, CA) and Oligo dT primers (Invitrogen, Carlsbad, CA). For rPCR, 2 μl of RT product (total cDNA) was amplified using SYBR Green PCR Mastermix (Applied Biosystems, Warrington, UK) under the following conditions: initial denaturation for 10 minutes at 95 °C, followed by 40 cycles consisting of 15 seconds at 95 °C and 30 s at 60 °C. Fold changes were calculated using the 2^−ΔΔCT^ method, considering glyceraldehyde 3-phosphate dehydrogenase (GAPHD) as reference mRNA^27^.

### Electrophysiology

Patch-clamp experiments were performed in the whole-cell configuration in voltage clamp mode, at room temperature. Current recordings were acquired using a personal computer connected to the EPC-9 amplifier (HEKA, Lambrecht, Germany). Holding potential was 0 mV unless otherwise indicated. Currents were recorded continuously over time and recordings were interrupted when current reached steady state levels (after thapsigargin application) to applied a single continuous voltage ramp from −100 to + 100 mV. Patch electrodes were prepared from borosilicate glass 1.5 mm internal diameter (World Precision Instruments). Pipette resistances range from 3–5 MΩ when filled with the pipette solution. Intracellular (pipette) solution contained (in mM): 110 K aspartate, 30 KCl, 1 MgCl2, 10 HEPES, 5 EGTA, and 5 Mg ATP, pH 7.4 adjusted with KOH (300 ± 3 mosM). The bath (extracellular) solution contained (in mM): 135 NaCl, 2.0 CaCl2, 1.17 MgSO4, 4.7 KCl, 5 dextrose, and 10 HEPES (300 mosM, pH 7.4). All voltages were corrected for liquid-junction potentials between external and internal solutions, as previously described^28^. Capacitive currents and series resistance were determined and corrected before each voltage ramp using the automatic capacitance compensation of the EPC-9. Leak subtraction was corrected from current ramps in the absence of thapsigargin or agonists. Current reported is pA/pF to compensate for differences in cell membrane areas. Data was analyzed and plotted with Igor Pro version 6 (Wavemetrics, OR).

### Confocal microscopy and TIRFM

To switch between TIRFM and confocal micropscopy in real-time, we utilized the lightguide-based Total Internal Reflection Fluorescence Microscopy apparatus (lg-TIRFM, TIRFLabs, Inc., Cary, NC)^29^. The lg-TIRFM system was mounted on an inverted IX81 Olympus microscope equipped with a high numeric aperture (1.45), 60X oil-immersion PlanApo objective (Olympus, Japan). The microscope was part of a FV1000 confocal microscope (Olympus, Japan). Using this configuration we were able to alternate between multicolor TIRFM and confocal microscopy in seconds. lg-TIRFM mode was utilized to explore STIM1 puncta assembly at areas closer to the plasma membrane (typically less than 100 nm, refer to^29^, for a detail explanation on the optical scheme of lg-TIRFM). To evaluate STIM1 puncta at greater depths into the cell cytosol, we switch to confocal microscopy. Using the lg-TIRFM system, the same cell is observed in TIRFM and confocal modes. This is feasible because switching between the two modes implies only changing the excitation source.

For co-localization studies under TIRFM we took advantage of the multicolor mode of lg-TIRFM to rapidly (milliseconds) switch among several excitation wavelengths, using a solid-state, LED-based multicolor illuminator MCI-7000 (TIRFLabs, Inc., Cary, NC). The excitation wavelengths used for the different florescent proteins were: YFP (405 nm) and CFP (455 nm). Emissions were collected using high-quality emission filters from chroma (Bellows Falls, VT). Pearson co-localization coefficients where calculated from co-localization channels between the different fluorescent proteins used in this study. Co-localization analysis was conducted using Imaris 8 (Bitplane, Switzerland). Co-localization analysis for both confocal and TIRFM images was conducted using the Pearson correlation coefficient, as it has been shown to provide more reliable results than the Mander’s overlap coefficient since the intensity of the fluorescence does not affect the outcome^30^.

### Intraluminal free calcium measurements

Endoplasmic reticulum (ER) intraluminal free calcium determinations were conducted using Mag-Fluo-4 (Molecular Probes, Life Technologies) as previously described^31^. Briefly, HEK293T cells were loaded with 2 μM of AM-ester derivative of Mag-Fluo-4 for 1 hour at 37 °C in saline solution without serum (in the dark). After the incubation cells were rinsed twice with saline solution and mounted on a sapphire cuvette for cell population studies using an Aminco-Bowman series 2 spectrofluorometer. The excitation wavelength was maintained at 485 nm and emission was collected at 515 nm. Mag-Fluo-4 fluorescence signal was normalized using the resting level (F/Fo) calculated during the initial 5 min recording period. To compare the different procedures, the area under the curve for Mag-Fluo-4 fluorescence change (F/Fo) is reported also.

### Co-immunoprecipitation (Co-IP) studies

HEK293T cells harvested from four 15 cm culture dishes were centrifuged and pelleted. Cell pellet was dissolved into the solution for protein isolation (Pierce, Rockford, IL). For co-immunoprecipitation studies, protein complexes were immunoprecipitated with anti-GFP antibody and a secondary antibody attached to magnetic beads (MagnaBind Beads, Pierce) as previously described^32^. In all experiments between 8–10 μg of total protein were loaded into each gel lane. For the identification of STIM1 and IP3R proteins, 6% acrylamide gels were casted, because of the very large molecular weight of IP3R. Due to the very large differences in molecular weights between IP3R (≈250 kDa) and STIM1 (90 kDa), the same immunoprecipitation samples were analyzed on different electrophoresis gels. After electrophoresis ended, the gel was transferred into nitrocellulose membranes using a semi-dry chamber from Biorad (Hercules, CA), following manufacturer instructions. Proteins were identified with their specific antibodies in western blot studies.

### Data and image analysis

All acquired images were analyzed using Imaris 8 (bitplane, Switzerland). Amira 6 (ThermoFisher Scientific, Hillsboro, Oregon) was used to plot height plots from STIM1 puncta.

Data were analyzed by one-way ANOVA followed by Bonferroni multiple comparisons tests or by two-tailed Student’s t-test (GraphPad Prism, GraphPad Software Inc). Unless otherwise indicated data are presented as means ± s.d. with significance set at ***p < 0.001, **p < 0.01 or *p < 0.05.

## Electronic supplementary material

Video 1

Video 2

Video 3

Video 4

Supplementary data
