# Supplementary material for: Association of the IP3R to STIM1 provides a reduced intraluminal calcium microenvironment, resulting in enhanced store-operated calcium entry
Source: Sci Rep. 2018 Sep 5;8:13252. doi: 10.1038/s41598-018-31621-0 (PMC6125598; doi:10.1038/s41598-018-31621-0)
Supplement: Supplementary file 5 — Supplementary data [file 41598_2018_31621_MOESM5_ESM.docx]

Association of the IP3R to STIM1 provides a reduced intraluminal calcium microenvironment, resulting in enhanced store-operated calcium entry

Alicia Sampieri^1^, Karla Santoyo^1^, Alexander Asanov^2^ and Luis Vaca^1*^

SUPPLEMENTAL INFORMACION

Figure 1

Figure 2

Figure 3

Figure 4


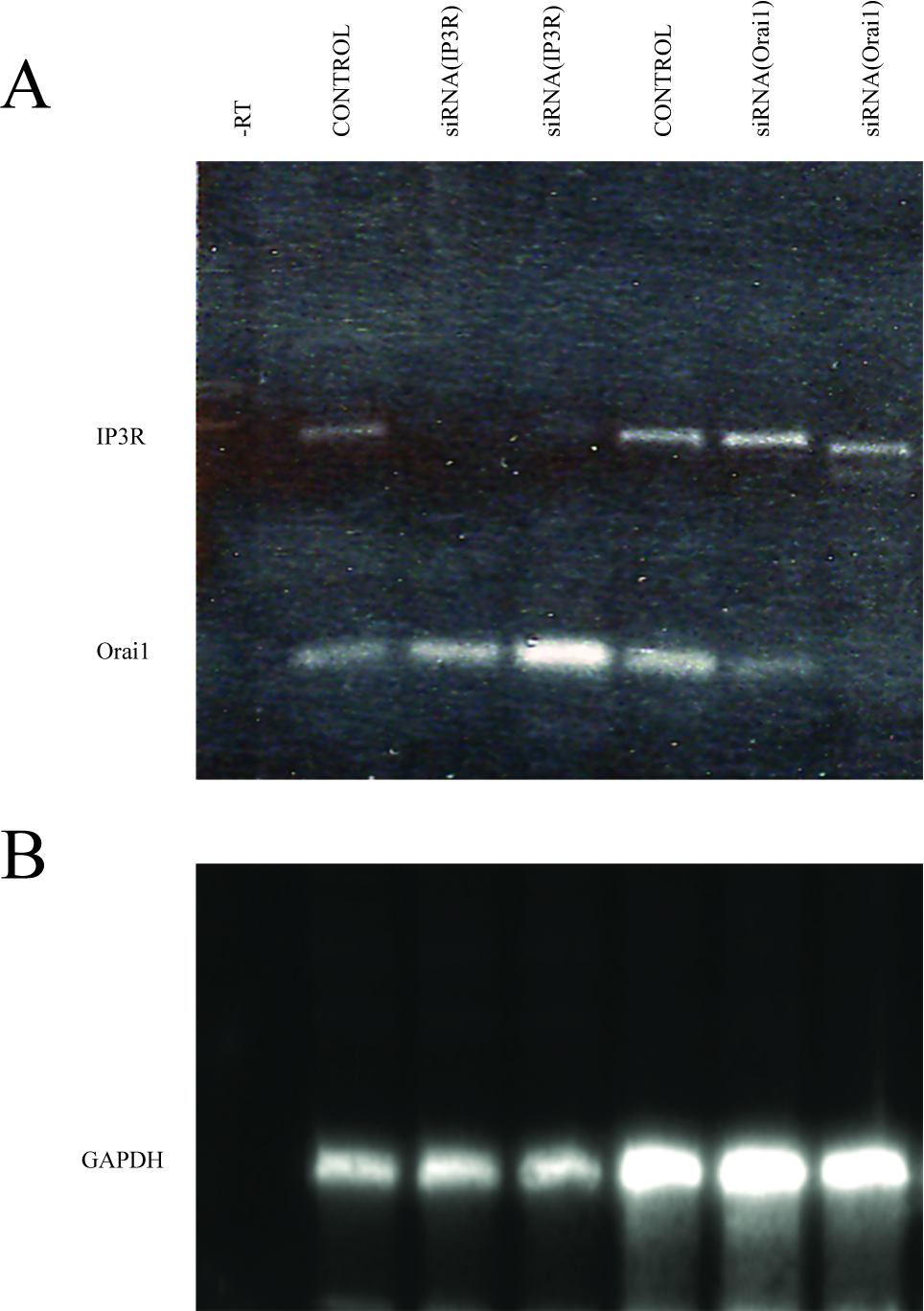
Figure 5

***Supplemental figure 1. Representative western blot gels***. For the identification of STIM1 and IP3R proteins, 6% acrylamide gels were casted, because of the very large molecular weight of IP3R. Due to the very large differences in molecular weights between IP3R (≈250 kDa) and STIM1 (90 kDa), the same immunoprecipitation samples were analyzed on different electrophoresis gels. After electrophoresis ended, the gel was transferred into nitrocellulose membranes using a semi-dry chamber from Biorad (Hercules, CA), following manufacturer instructions. Proteins were identified with their specific antibodies in western blot studies.

***Supplemental figure 2. Time courses for whole-cell currents activated by agonists and TG***. (**A**) Time course of the current activation by bradykinin (BK) and thapsigargin (TG) in HEK293 cells. Plots show the current density (mean ± standard deviation from at least 17 cells. (**B**), representative whole-cell currents elicited by a ramp from -100 to +100 mV obtained in 500 milliseconds. The numbers above the currents show the times at which the representative currents were obtained (1,2,3 encircled). In green are shown the currents elicited with bradykinin (BK) and in red with thapsigargin (TG).

***Supplemental figure 3. Different agonists coupled to the IP3 cascade increase SOCE when IP3R is overexpressed.*** Typical whole-cell currents in resting conditions (grey) and after stimulation with thapsigargin (TG, green) and histamine (His, in red) in control cells (**A**) and cells overexpressing IP3R (**B**). Whole-cell currents in resting conditions (grey) and after stimulation with thapsigargin (TG, green) and carbachol (Car, in red) in control cells (**C**) and cells overexpressing IP3R (**D**). **E**, whole-cell current density for all the conditions mentioned above with control cells and cells overexpressing IP3R. Data represents the mean ± standard deviation of at least 20 independent measurements for each condition.

***Supplemental figure 4. A large portion of the endogenous whole-cell current is produced by Orai1.*** A, whole-cell patch clamp currents elicited with a ramp from -100 to + 100 mV. Control represents cells activated with bradykinin. Cells transfected with siRNA directed to knowkc down Orai1 (siRNA(Orai1)) or a scramble sequence (siRNA(Scramble)) used as negative control. **B**, whole-cell current density for all the conditions mentioned above. Data represents the mean ± standard deviation of at least 20 independent measurements for each condition. **C**, representative intracellular calcium measurements using Fura-2 in cell populations under control conditions (no siRNA used), cells transfected with the siRNA(Oai1), cells transfected with siRNA(scramble) and cells transfected with siRNA(IP3R). **D**, relative area obtained from the experiments shown in C. Data represents the mean ± standard deviation of at least 10 independent measurements for each condition. **E**, RT-PCR directed to evaluate the reduction in mRNA for endogenous Orai1 and IP3R. RT-PCR reactions were carried in parallel to determine amounts of mRNA for Orai1 and IP3R and in the second reaction tube GAPDH. The two lines showing siRNA(IP3R) represent mRNAs isolated from two independent RNAi experiments. Gels were run separately for Orai1-IP3R and GAPDH as shown in the figure. Control represents cells not treated with any siRNA. GAPDH was used as control, as previously reported ^1^. Only a portion of the gels is shown in the figure, full-length gels are illustrated in supplementary figure 5.

***Supplemental figure 5. Representative DNA blot gels from RNAi experiments***. Scans from the entire agarose gels where the different RT-PCR samples were run (see supplementary figure 4E). **A**, scans from the gel identifying Orai1 and IP3R. **B**, scans from the gel identifying GAPDH.

***Supplemental video 1.*** Time course of the recruitment of IP3R (shown in red) to puncta. STIM1-CFP is shown in green. Video acquired using TIRFM. The duration of the video is 3 minutes. Time of bradykinin application is shown.

**Supplemental video 2.** Reduction of fluorescence signal from STIM1-GCaMP upon bradykinin (Bk) and thapsigargin (TG) stimulations in cells monitored using TIRFM. TG or Bk was applied to induce puncta formation and fluorescence was monitor for 300 seconds. For illustration purposes fluorescence intensity was mapped as height, according to the pseudo color scale shown in the video (blue less fluorescence and red more fluorescence).

**Supplemental video 3.** Time courses of changes in fluorescence reported by the sensor STIM1-GCaMP (left panel) and ER-GCaMP (right panel). Cell was stimulated with bradykinin 100 nM.

**Supplemental video 4.** Time course of STIM1 puncta formation and FRET signal between IP3R and STIM1. The height plot shows the STIM1-CFP fluorescence intensity (shown in red) and the surface plot shows the FRET between STIM1-CFP and YFP-IP3R (shown in white above the height plot). Notice that the FRET signal increases after STIM1-CFP is partially formed, suggesting that IP3R incorporates into the STIM1 puncta later during puncta formation.

REFERENCES

1. Moreno, C., Sampieri, A., Vivas, O., Pena-Segura, C. & Vaca, L. STIM1 and Orai1 mediate thrombin-induced Ca(2+) influx in rat cortical astrocytes. *Cell Calcium* **52,** 457–467 (2012).
